# Transition probabilities between changing sensitization levels, waitlist activity status and competing-risk kidney transplant outcomes using multi-state modeling

**DOI:** 10.1371/journal.pone.0190277

**Published:** 2017-12-29

**Authors:** Sanjay Kulkarni, Isaac Hall, Richard Formica, Carrie Thiessen, Darren Stewart, Geliang Gan, Erich Greene, Yanhong Deng

**Affiliations:** 1 Department of Surgery, Section of Organ Transplantation & Immunology, Yale School of Medicine, New Haven, CT, United States of America; 2 Department of Medicine, Section of Nephrology, Yale School of Medicine, New Haven, CT, United States of America; 3 Department of Medicine, Section of Nephrology, University of Utah School of Medicine, Salt Lake City, UT, United States of America; 4 United Network for Organ Sharing, Richmond, VA, United States of America; 5 Yale Center for Analytical Sciences, Yale School of Public Health, New Haven, CT, United States of America; University of Toledo, UNITED STATES

## Abstract

**Background:**

Sensitization and activity status are associated with kidney transplant waitlist mortality. Unknown is how changes in these covariates after listing impact transplant outcomes.

**Methods:**

Two cohorts were created from the OPTN (Organ Procurement and Transplantation Network) database, one pre-KAS (new kidney allocation system) (10/01/2009-12/04/2013, n = 97,793) and one post-KAS (12/04/2014-06/17/2015, n = 13,113). Multi-state modeling provides transition probabilities between intermediate states (CPRA category/activity status combinations) and competing-risk outcomes: transplant (living), transplant (deceased), death, or other/well.

**Results:**

Transition probabilities show chances of converting between intermediate states prior to a competing-risk outcome. One year transplant probabilities for post-KAS candidates with a CPRA of 0%(P, 0.123[95% CI, 0.117,0.129]), 1–79%(P, 0.125 [95% CI, 0.112,0.139]), 95–98%(P, 0.242[95% CI, 0.188, 0.295]) and 99–100%(P, 0.252 [95% CI, 0.195, 0.308]) were significantly higher than the pre-KAS cohort; they were lower for CPRA 80–89%(P, 0.152 [95% CI, 0.116,0.189]) and not statistically different for CPRA 90–94%(P, 0.180 [95% CI, 0.137,0.223]) candidates. Post-KAS, Whites had a statistically higher transplant probability only at a CPRA of 99–100%.

**Conclusion:**

Multi-state modeling provides transition probabilities between CPRA/activity status combinations, giving estimates on how changing patient characteristic’s after listing impact outcomes. Preliminarily, across most CPRA categories, there was no statistical difference in transplant probabilities between Whites, Blacks and Hispanics following KAS implementation, however, this finding requires longer follow-up for validation.

## Introduction

Statistical models that account for changing patient-level factors over time enhance the ability to provide information to clinicians and patients regarding how specific clinical changes impacts clinical outcomes [1]. This represents an unmet need in OPTN/UNOS (Organ Procurement and Transplantation Network/United Network for Organ Sharing) waitlist data analyses, which are used to determine outcomes for patients listed for kidney transplant, yet often do not account for patient characteristics that change after initial listing [2].

The process from listing to outcome can be modeled as a continuous time-stochastic process, where the inclusion of patient characteristics that change after initial listing are essential to determining how these differences affect waitlist outcomes. Amongst the variables that impact waitlisted patients, activity status to receive organ offers (designated as active vs. inactive) and the level of antibody sensitization (designated by Calculated Panel Reactive Antibodies (CPRA)) are both associated with increased waitlist mortality [3, 4]. CPRA may increase due to a specific sensitization event, but there are no estimates on the probability of this event which limits how clinicians discuss risks, like blood transfusions, with waitlist candidates. Prior to the implementation of the new Kidney Allocation System (KAS), candidates with a very high CPRA, who tend to be incompatible with most donors, had a lower likelihood of receiving deceased donor organ offers, which is a recognized factor contributing to less access to kidney transplant for underserved populations [5, 6].

KAS (OPTN policy 8.3; effective December 4, 2014) was designed to improve transplant rates of highly sensitized patients and address known disparities in access to transplantation. Because of sensitization events or clinical status changes, CPRA or waitlist activity status may change over time. Although most OPTN/UNOS data analyses have treated these variables as fixed covariates, based on data proximate to the date of listing, how these variables change over time are essential to understanding the true impact on waitlist outcomes [4, 7]. As these outcomes are used to formulate policy changes used in organ allocation and for educating patients on the waitlist, the ability to provide probabilities, or true percentages versus odds or relative risks, enhances the understanding of how the changes affect waitlist outcomes.

It is recognized that transplant outcomes for waitlisted patients should be considered as competing risks [8]. A waitlisted patient might experience one of a few events such as transplantation (living or deceased), death, getting well, or removal from the waitlist for other reasons. Conventional methods, such as Kaplan-Meier survival analysis (1-KM), do not properly account for these competing risks, since right-censoring competing-risk events overestimates the probabilities for the primary event of interest [8]. Furthermore, analyses focusing on the association of risk factors and cause-specific hazard cannot be extended to probability or cumulative incidence function in the presence of competing risks [9]. Hart et al. used competing-risk modeling to provide probability of outcomes for patients listed for kidney transplant [8]. However, this model used CPRA as a fixed covariate and did not utilize activity status in a time-dependent manner. Sapir-Pichhadze et al. conducted an analysis where CPRA was used as a time-varying covariate with implications on cardiovascular mortality [3]. However, this analysis did not consider activity status and also did not evaluate the impact of CPRA on the probability of competing waitlist outcomes. Grams et al. have shown that activity status is associated with waitlist mortality by using a Cox-proportional hazards model with activity status treated as a time-varying covariate; however, the association of transplant outcomes with time-varying activity status was not studied because inactive participants could not receive a deceased donor organ [4]. It is important to recognize the necessity of including activity status in competing-risk models, as inactive patients are not at risk for deceased donor transplants, but are at risk for other competing outcomes, such as living donor transplants, death and waitlist removal due to other reasons, all of which regular COX regression models fail to reconcile.

From the disease/recovery process point of view, a waitlisted patient might experience different levels of sensitization or have changes in their waitlist status before experiencing a clinical endpoint, such as transplant or death. Multi-state modeling, which is an extension of competing-risk models, provides a framework that permits analysis of this type of event history data [10, 11]. Differing CPRA levels and activity status combinations are treated as transient or intermediate states, while the transplant outcomes are considered as absorbing states. Forthcoming, we present our multi-state modeling of CPRA level and activity status using the OPTN/UNOS database, which is a novel approach to understand how patients transition between differing CPRA/activity state combinations, how these transitions impact probabilities of competing-risk outcomes, and if KAS resulted in improved access to deceased donor transplant to underserved populations.

## Materials and methods

### Study population

The Yale University Institutional Review Board approved of this study. Retrospective cohorts of adult (age>18) first-time registrants for kidney transplant were created from the OPTN/UNOS database. The first cohort included pre-KAS candidates (10/01/2009-12/04/2013), while the second cohort included candidates registered post-KAS (12/04/2014-06/17/2015). Intervals were used to ensure at least one year follow-up for patients in both cohorts (Panel A in S1 Fig). Registrants listed for another organ, or those dually listed for pancreatic islets were excluded. Additionally, our preliminary analysis showed significant variation in listing practices for those candidates that were listed at multiple centers. For this reason, we chose to only include primary center data for multiply listed candidates. Because this analysis used two cohorts in a before/after design, we included first-time registrants only to decrease confounding for kidney transplant candidates who may have experienced an outcome in both time-limited cohorts.

### Multi-State model construction

When time-varying covariates are included in the modeling process, estimating cumulative incidences and survival probabilities is no longer feasible [9]. Multi-state modeling represents a series of nested competing risks [12], therefore, transition probabilities to any absorbing states may be estimated at any time (t) in the disease/recovery process from any initial or intermediate state (Panel B in S1 Fig).

Though CPRA is a continuous measure ranging from 0% to 100%, practical limitations of multi-state modeling required grouping of CPRA into a finite number of assigned categories: 0%, 1–79%, 80–89%, 90–94%, 95–98%, and 99–100%. These groups were chosen based on clinical, statistical, and allocation policy considerations. For example, prior to KAS, candidates with CPRA of 80% or greater received priority points in allocation. After KAS, priority points are awarded on a sliding scale to reflect the inherent nonlinearity in the relationship between CPRA and access to compatible kidney donors, which is especially evident for CPRA values exceeding 90%. In addition to priority points, candidates with CPRA of 99 or 100% receive regional and national priority, respectively [13].

**Fig 1:**
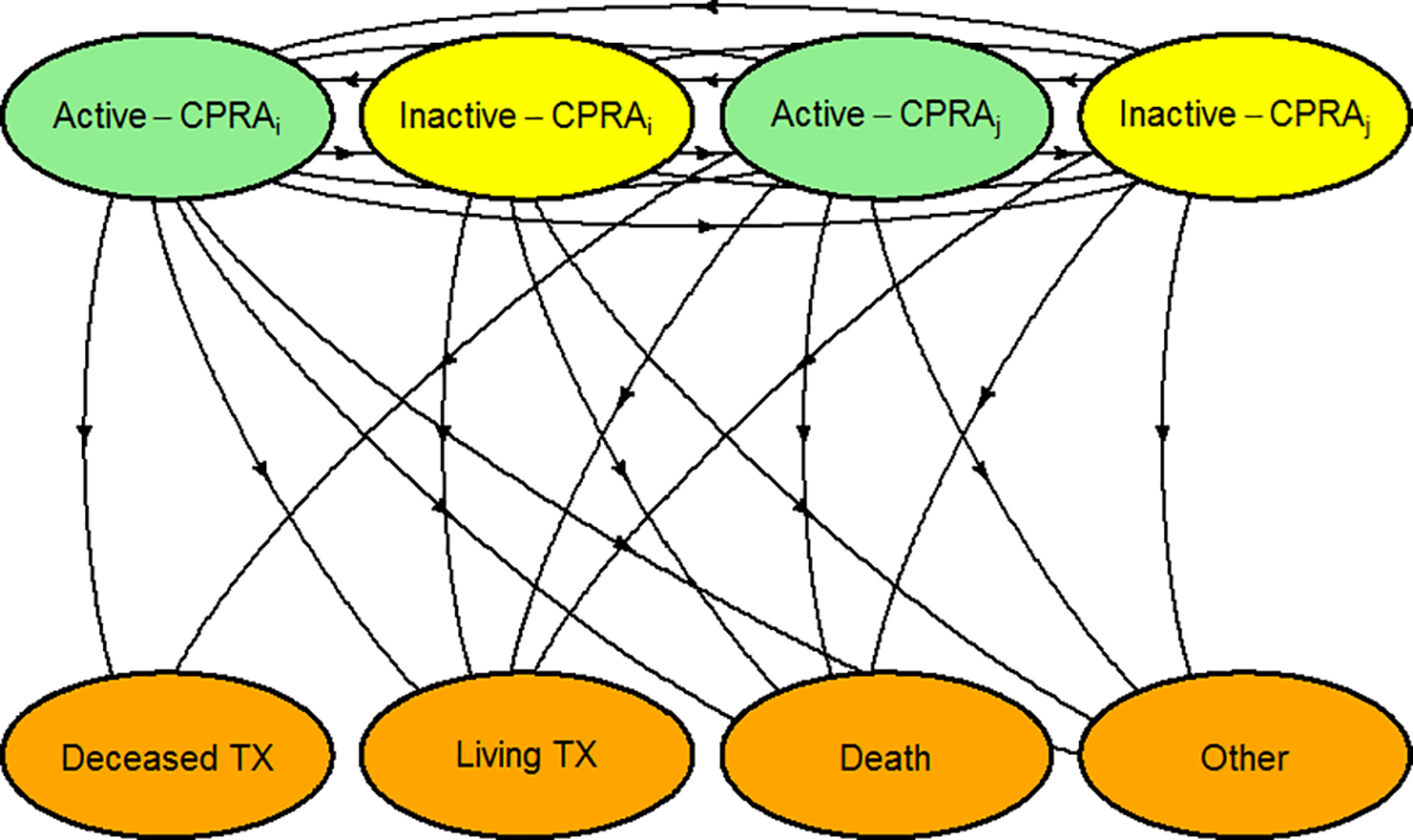
Multi-state modeling framework. CPRA i (i = 1 to 6) is one of the six CPRA categories: 1) 0%; 2) 1–79%, 3) 80–90%, 4) 90–95% 5) 95%-98%, 6) 99%-100%). CPRA j (j = 1 to 6) is another CPRA category, different from category i. There are 12 initial/intermediate states. (see supplemental materials for additional details).

Combinations of 6 CPRA categories and two waitlist statuses led to 12 initial states. Outcomes were classified into 4 categories and used as the absorbing states: deceased donor transplant; living donor transplant; death or removal due to deteriorating medical condition; or removal due to other reasons. The model contains a total of 174 possible transitions. Transitions from inactive states to deceased donor transplant were not modeled, since inactive candidates are not eligible for deceased donor transplant. Candidates enter the model in any of the 12 initial states and may transition repeatedly between these states before reaching an absorbing state (Fig 1).

### Statistical analysis

Baseline demographic candidate characteristics were compared between pre- and post-KAS cohorts. Standardized differences between the two cohorts were calculated using Cohen’s d [14].

Cumulative transition-specific hazards were estimated non-parametrically using the Nelson-Aalen estimator. The probabilities of transition were estimated using the Aalen-Johansen estimator. Transition probability, usually involving two states (e.g. health and death) and two time points, combines both direct and indirect transitions from one state to another (Panel C in S1 Fig). Standard errors associated with cumulative transition hazards and transition probabilities were estimated using Greenwood methods [15]. This multi-state model represents a time-inhomogeneous Markov, meaning the future transition out of a given state is dependent on the past only through elapsed time from origin.

Transplant probabilities were estimated separately in pre- and post-KAS cohorts. Since the follow-up time for some patients in pre-KAS cohort straddled two eras, outcomes for candidates listed pre-KAS were censored at KAS implementation to exclude the impact of KAS on the estimation of probabilities in the pre-KAS cohort. Second, to assess if KAS had an impact on candidates listed pre-KAS, who did not experience an outcome in the pre-KAS era, we lifted right-censoring at KAS implementation and continued to measure their outcomes in the post-KAS period (Panel A in S1 Fig). The rationale for this is to utilize the assumption of independent right-censoring, which assumes that the sample observed after right-censoring is representative of the population without censoring. Therefore, if KAS truly had an effect–if pre-KAS patients who had events in the post-KAS era can’t be represented by patients who had events in the pre-KAS era–we would expect that probabilities of deceased donor transplant for highly sensitized pre-KAS candidates after one year on the waitlist would be higher than the probabilities estimated by censoring events at KAS implementation.

Data management was conducted using SAS software, version 9.4 of the SAS System for Windows (Cary, NC, USA) and multi-state modeling analysis was performed in R.3.1.0 using the mstate package [16]. Two-sided p-values less than 0.05 were considered statistically significant.

## Results

### Study population

There were 97,793 and 13,113 adult registrants in pre- and post-KAS cohorts, respectively. The two cohorts were very well matched on baseline demographics, with negligible standardized difference (from 0.0053 to 0.0555) [15] and there were no statistically significant differences in initial CPRA category between the cohorts (Table 1).

**Table 1:** Pre-KAS and Post-KAS cohort demographics.

	Pre-KAS(N = 97793)		Post-KAS(N = 13113)		
	Count	%	Count	%	*Standardized difference*
Age					0.0343
18–39	16139	16.5	2295	17.5	
40–64	62804	64.2	8289	63.2	
≥65	18850	19.3	2529	19.3	
Gender					0.0053
Male	59960	61.3	8273	63.1	
Female	37833	38.7	4840	36.9	
ABO					0.01
O	47033	48.1	6254	47.7	
A	32804	33.5	4410	33.6	
B	14025	14.3	1905	14.5	
AB	3931	4.0	544	4.2	
Race					0.0555
White	43539	44.5	5677	43.3	
Black	28304	28.9	3657	27.9	
Hispanic	17649	18.1	2564	19.6	
Asian	6275	6.4	955	7.3	
Other	2026	2.1	260	2.0	
ESRD Diagnosis					0.0488
Diabetes	35000	35.8	4771	36.2	
Glomerulonephritis	10971	11.2	1436	11.0	
Graft Failure	1950	2.0	265	2.0	
Hypertension	22962	23.5	2841	21.7	
Other	26910	27.5	3800	29.0	
PRA Category					0.0231
0–79%	95925	98.1	12884	98.25	
80–89%	575	0.59	56	0.43	
90–94%	356	0.36	45	0.34	
95–98%	443	0.45	62	0.47	
99–100%	494	0.51	66	0.50	

### Transition probabilities among cPRA States at one year post-listing

**Fig 2:**
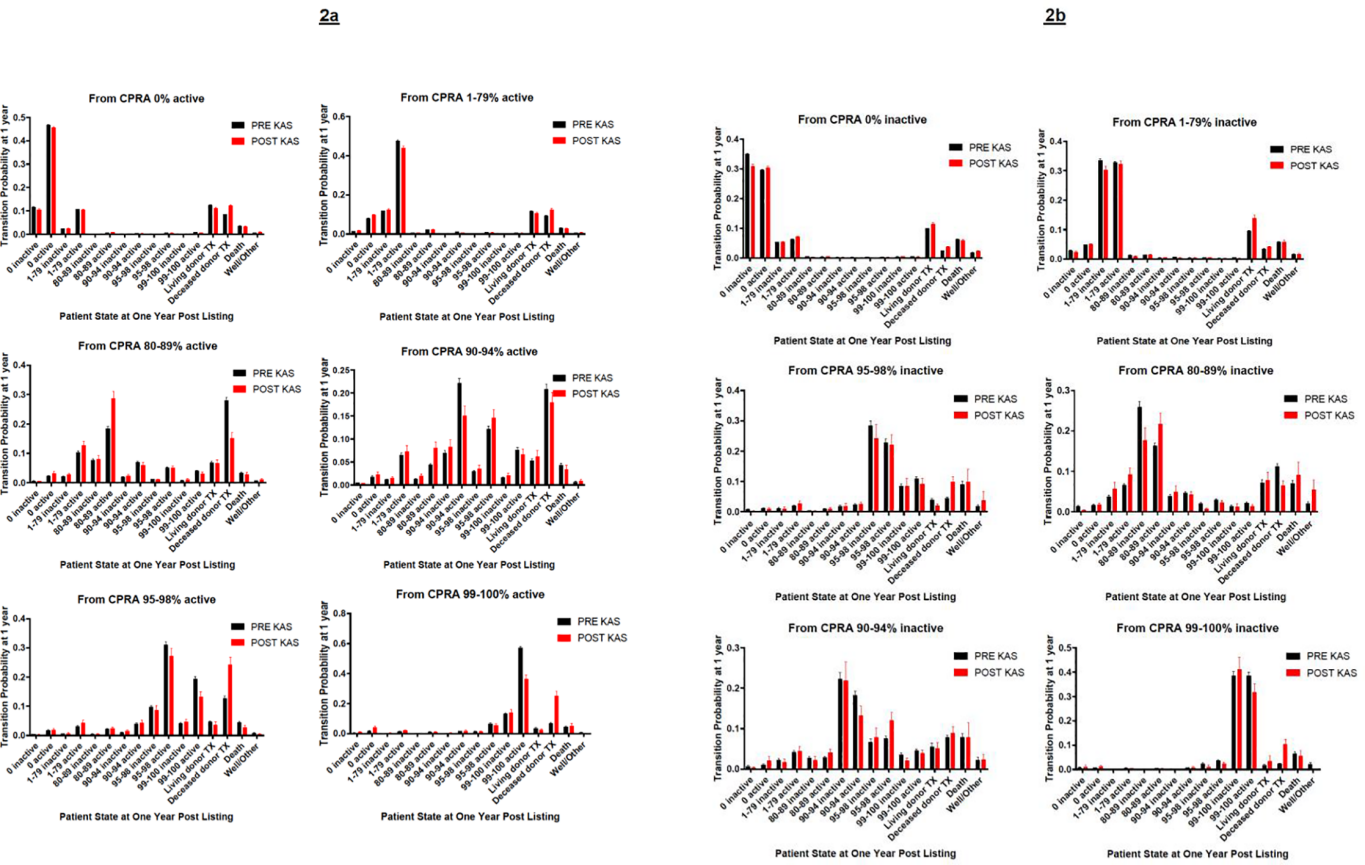
Cross sectional plots of transition probabilities among states at one year post-listing. **(Panel A)** Cross-sectional plots showing transition probabilities from initially active CPRA states. **(Panel B)** Cross sectional plots showing transition probabilities from initially inactive CPRA state.

Fig 2A and 2B provide cross sectional plots of transition probabilities through one year after listing for both cohorts, given initial activity status and CPRA category at the day of listing (day 0). Individuals are at risk of moving from these initial states to another state (x-axis), as indicated by varying transition probabilities for each state or outcome at one year (y-axis). These plots help to understand the time-varying nature of transitioning between intermediate states and how these changes impact competing patient outcomes. After one year of listing, patients who were active at day 0 were more likely to stay active in their initial CPRA categories. Patients who were inactive at day 0 were more likely to remain in their original CPRA categories and equally likely to change to active status or remain inactive. These plots show that if patients with CPRA of 0% or 1–79% at day 0 were to leave their initial CPRA categories in the first year, they would be most likely to transition between these two states rather than transition to a higher CPRA. Individuals with a CPRA of 80–89% would be more likely to transition to a CPRA of 1–79%, 90–94%, 95–98% or 99–100%, and those with a CPRA of 95–98% would be more likely to transition to a CPRA of 99–100%. Finally, individuals leaving the 99–100% CPRA category would be more likely to transition to a CPRA of 95–98%.

### Activity status and mortality

**Fig 3:**
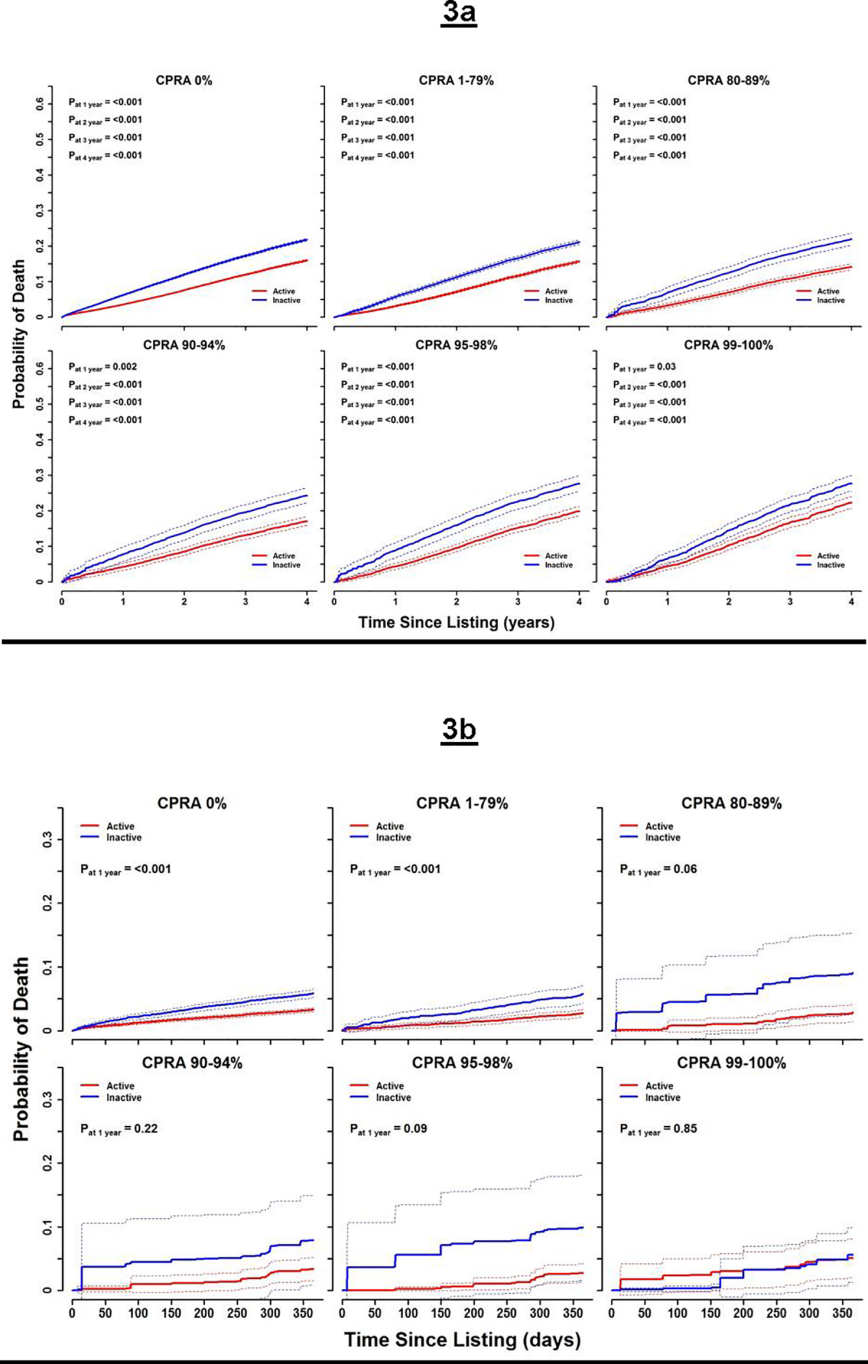
Cumulative probability of death stratified by activity status. **(Panel A)** Line graphs of individuals listed active vs. inactive in the pre-KAS cohort. **(Panel B)** Line graphs of individuals listed active vs. inactive in the post-KAS cohort.

Fig 3A shows the probabilities of death predicted from initial CPRA category and activity status for the pre-KAS cohort. Across all initial CPRA categories, candidates listed inactive at day 0 had significantly higher probabilities of death even though they might have transitioned to active status at some point during the year (S1 Table shows probability estimates). Our dynamic prediction analysis confirms the association of inactive status and death, as candidates inactivated anytime within one year of listing had an increased probability of death at year-3 (S2 Fig and S2 Table). Analysis from the post-KAS cohort show a similar trend (Fig 3B).

### Impact of KAS on deceased donor transplant probabilities

**Fig 4:**
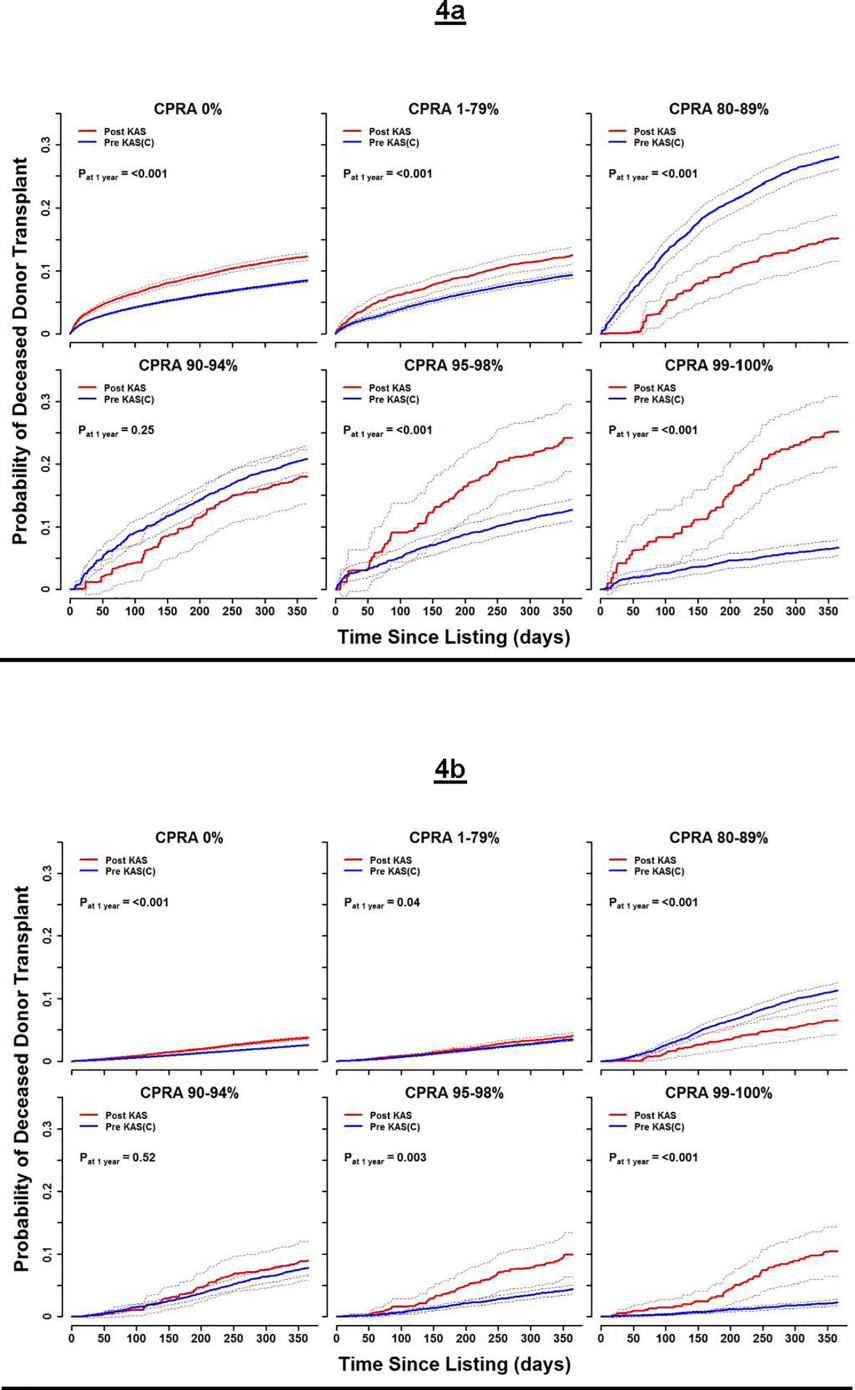
Pre-KAS versus Post-KAS transplant probabilities. **(Panel A)** Line graphs with 95% CIs comparing the probabilities of deceased donor transplants between the pre-KAS and post-KAS cohorts, stratified by initially-active CPRA state. **(Panel B)** Line graphs with 95% CIs comparing the probabilities of deceased donor transplants between the pre-KAS and post-KAS cohorts, stratified by initially-inactive CPRA state.

Fig 4A (active status) and 4B (inactive status) show the probabilities of receiving a deceased donor transplant, predicted from initial listing CPRA category and activity status. Individuals listed active post-KAS with a CPRA of 0–79% and 95–100% had a significantly higher transplant probability within the first year of listing, compared to the similar pre-KAS CPRA groups (S3 Table shows probability estimates). Compared to the pre-KAS cohort, individuals listed post-KAS with an active CPRA of 80–89% had a statistically lower transplant probability within the first year of listing. Although individuals in the active CPRA 90–94% group had a lower probability of transplant following KAS implementation, this difference did not reach statistical significance. The probabilities of deceased donor transplant predicted from the initial inactive states were much lower than those from the active states, since inactive patients must first transition to an active state to receive an organ offer. Still, the same pre- versus post-KAS patterns manifested for initially inactive patients, with a sharply higher transplant probability post-KAS for initially inactive patients with CPRA 99–100% (Fig 4B). After lifting right-censoring for the pre-KAS cohort at KAS implementation, the probabilities of transplant for patients with an initial CPRA of 95–100% after one year increased, compared to probabilities obtained with censoring (S3 Table).

### Racial subgroup analysis

Analyses on initial actively waitlisted pre-KAS patients demonstrated that Whites had a higher probability of deceased donor transplant compared to Hispanics or Blacks across all initial CPRA levels (Panel A in S4 Fig). Black candidates with initial CPRA 0–79% were more likely to be transplanted than Hispanic candidates, though this trend did not hold for higher initial CPRA categories. Post-KAS analysis of initially actively listed patients showed no significant advantage for Whites across all initial CPRA categories, though they may retain some advantage at a CPRA of 99–100% (Panel B in S4 Fig). Further study with longer follow-up time and a larger sample needs to be performed to validate this finding.

## Discussion

This study provides the first multi-state modeling of OPTN/UNOS data and focuses on how waitlist candidates change sensitization levels and activity status following listing with the impact of these changes to transplant competing-risk outcomes. We also explore the effect of KAS on competing-risk transplant outcomes using this new methodology. Studies to date that have looked at differences in the allocation of kidney transplants have used statistical approaches that generally provide hazard rates, odds ratios and relative risks to determine the effect of predictor variables on transplant rate of a specific cohort. These analyses have been the central way that OPTN/UNOS data has been used to measure outcomes and, importantly, to shape allocation policy [13]. Multi-state modeling provides information that is clinically useful regarding actual probabilities, as opposed to relative risk of transplant outcomes, while simultaneously accounting for time- varying covariates and competing risks. Importantly, this modeling technique provides a way to adjust for listing status as a time-varying covariate, which cannot be accommodated in traditional Cox proportional models because inactive patients are not at risk for deceased donor transplants, but are at risk for other competing outcomes, such as living donor transplants, death, and waitlist removal. Since listing status may change, the number of competing outcomes a patient can experience might change over time. Grams et al. have shown that initial active status at baseline is associated with lower rates of transplantation using modified Poisson regression [4]. However, this work acknowledged that the association of time-varying activity status within a competing risk analysis cannot be performed. Multistate modeling, creating transition states using time-varying covariates, solves this problem by removing transitions from inactive status to deceased-donor transplant. Probabilistic forecasting is likely to be particularly important in developing new strategies to address persistent geographic, demographic and other general clinical disparities in transplantation.

The estimated probabilities presented were all predicted from first day of listing. Transition probabilities to any state, at any future time, can be estimated from any predesignated state and time. For example, we provide the probability of deceased donor transplant outcomes at year-3 predicted timeframe from anytime within the first year of listing for pre-KAS cohort (S4 Table). This dynamic property of multi-state modeling provides a useful tool for patient education. For instance, given a patient listed as active with CPRA 0% at day 0, the predicted probability of deceased donor transplant at year-3 would be around 0.211 (21.1%), and if this patient were to transition to an inactive state with CPRA 1–79% at day 60, the predicted probability of deceased donor transplant at year-3 would decease to 0.124 (12.4%). To date, there have been no estimates on the probability of highly sensitized candidates moving to lower levels of sensitization or probabilities of how a sensitizing event may result in a change in CPRA status. Acknowledging that there is limited information regarding sensitizing events in the OPTN/UNOS database, the transition probabilities provided in this analysis may help better educate candidates regarding the risk of a sensitizing event or the chances sensitization level becoming lower at a specified time-period (1-year in this analysis) after listing.

This study shows that the probability of receiving a deceased donor transplant based on candidate CPRA has changed significantly with the implementation of KAS and supports early observations on its effects [2, 17, 18]. One of the priorities of KAS was to increase kidney transplants in individuals with a CPRA>98%, who previously experienced unusually long wait times. Although the observed statistically significant increases in transplant probability for candidates with CPRA 0% and 1–79% could be related to the overall increases in kidney transplants that occurred in the post-KAS timeframe [2, 17], the increase in kidney transplants in the two highest CPRA states likely came at the expense of the CPRA 80–89% group, which had a lower probability of deceased donor transplant probability following KAS.

Subgroup analysis on the pre-KAS cohort confirmed the known racial disparities in access to kidney transplant [19]. We provide additional characterization, stratified by CPRA category and activity status, with statistically significant differences in transplant probability observed between Whites and Blacks/Hispanics pre-KAS (Panel A in S4 Fig). Following KAS implementation, we did not find a significant advantage for Whites across all initial CPRA levels, though they may retain some advantage at higher initial CPRA levels (Panel B in S4 Fig). We speculate that HLA matching and the greater number of zero-mismatch transplants at the higher CPRA levels contributed to the advantage observed in the White CPRA 99–100% population [5].

This study was limited to variables that are included in the OPTN/UNOS database, and as a retrospective study, a true assessment of causality has limitations. For example, information on sensitizing events would have provided greater clinical relevance on how these events are associated with a transition probability to higher CPRA categories. There are several baseline demographic variables, such as blood type, geographic location (donor service area) and dialysis status that can impact transplant outcomes. The multi-state model presented in this analysis focused on two important variables, CPRA and activity status, that are known to be associated with waitlist mortality [3, 4]. With only these two variables, our model had 174 transitions, which limited our ability to then include other relevant baseline variables into the model. To estimate the probabilities of transplant outcomes that would include all relevant baseline demographic variables, our future analysis will focus on semi-parametric modeling, in which the effects of covariates are better evaluated by controlling for other confounders. To assure at least one year follow-up for the post-KAS cohort, we used a relatively short time-frame to evaluate the impact of KAS; therefore, the observed lack of racial disparity noted in most post-KAS CPRA groups requires a larger dataset with longer follow-up for validation. Furthermore, to examine the changes in racial disparities after KAS implementation, we performed subgroup analysis through our non-parametric model, relying on the fact that the distribution of baseline demographics was balanced pre- and post-KAS. Finally, there were noted changes in the number of kidney transplants following KAS implementation [2] and it is likely that listing practices may have changed in the post-KAS period, given the emphasis on dialysis time in kidney allocation [2]. It is not clear what the impact of these and additional differences in clinical practice patterns were on the probabilities that were observed between CPRA categories and the effect of these observations on our post-KAS analysis.

This study provides the first multi-state modeling of the OPTN/UNOS kidney transplant waitlist. Clinically, CPRA may increase due to sensitizing events, such as blood transfusions or pregnancies, and transplant centers may change the activity status of a waitlisted patient due to a variety of reasons, including medical unsuitability, incomplete work-up, or financial concerns. Transplant centers notify practitioners and patients of these changes, but have not been able to convey the granular implications of these changes on transplant outcomes. For example, a waitlist candidate that has been made inactive one year after listing will now have an estimate on what the impact of this change is to all candidates who experience a change to inactive status. The fact that this status change now represents a quantifiable increase in mortality should be communicated to candidates so shared decision-making care plans can be developed to improve their chances of obtaining a deceased or living donor transplant. Transition probabilities between different CPRA levels and changes in activity status over time show both the likelihood of these changes and their effect on transplant outcomes by providing probability data that can better educate practitioners and patients on the implications of changing clinical status while waiting for a deceased donor kidney transplant.

## Supporting information

S1 Fig (Panel A) Cohort Selection (Panel B) Multi-State Model can be Viewed as a Series of Nested Competing Risk Models (Panel C) Transition Hazard and Transition Probability.
(DOCX)

S2 Fig Dynamic prediction of the probability of death at year-3 in pre-KAS cohort. (DOCX)

S3 Fig Dynamic prediction of the probability of deceased donor transplant at year-3 in pre-KAS cohort. (DOCX)

S4 Fig (Panel A) Line Graphs with 95% CI Showing Probabilities of Deceased Donor Transplant Between Whites, Hispanics and Blacks, Stratified by CPRA Category for Actively Listed Pre-KAS Individuals (Panel B) Line Graphs with 95% CI Showing Probabilities of Deceased Donor Transplant Between Whites, Hispanics and Blacks, Stratified by CPRA Category for Actively Listed Post-KAS Individuals.
(DOCX)

S1 Table Probability of death with 95% CI predicted from initial status (Day 0 from listing). (DOCX)

S2 Table Dynamic prediction of the probability of death at year-3 in pre-KAS cohort, given disease history within year-1 of listing. (DOCX)

S3 Table Probability of deceased donor transplant with 95% CI predicted from day 0 of listing. (DOCX)

S4 Table Dynamic prediction of the probability of deceased donor transplant at year-3 in pre-KAS cohort, given disease history within first year of listing. (DOCX)

S1 Database (7Z)

S2 Database (7Z)

## Abbreviations

OPTN: Organ Procurement Transplantation Network
UNOS: United Network for Organ Sharing
