# Supplementary material for: Transition probabilities between changing sensitization levels, waitlist activity status and competing-risk kidney transplant outcomes using multi-state modeling
Source: PLoS One. 2017 Dec 29;12(12):e0190277. doi: 10.1371/journal.pone.0190277 (PMC5747475; doi:10.1371/journal.pone.0190277)
Supplement: S1 Fig — (Panel A) Cohort Selection (Panel B) Multi-State Model can be Viewed as a Series of Nested Competing Risk Models (Panel C) Transition Hazard and Transition Probability. (DOCX) [file pone.0190277.s001.docx]

**Supplemental information**

**Panel A S1 Fig. Cohort Selection**


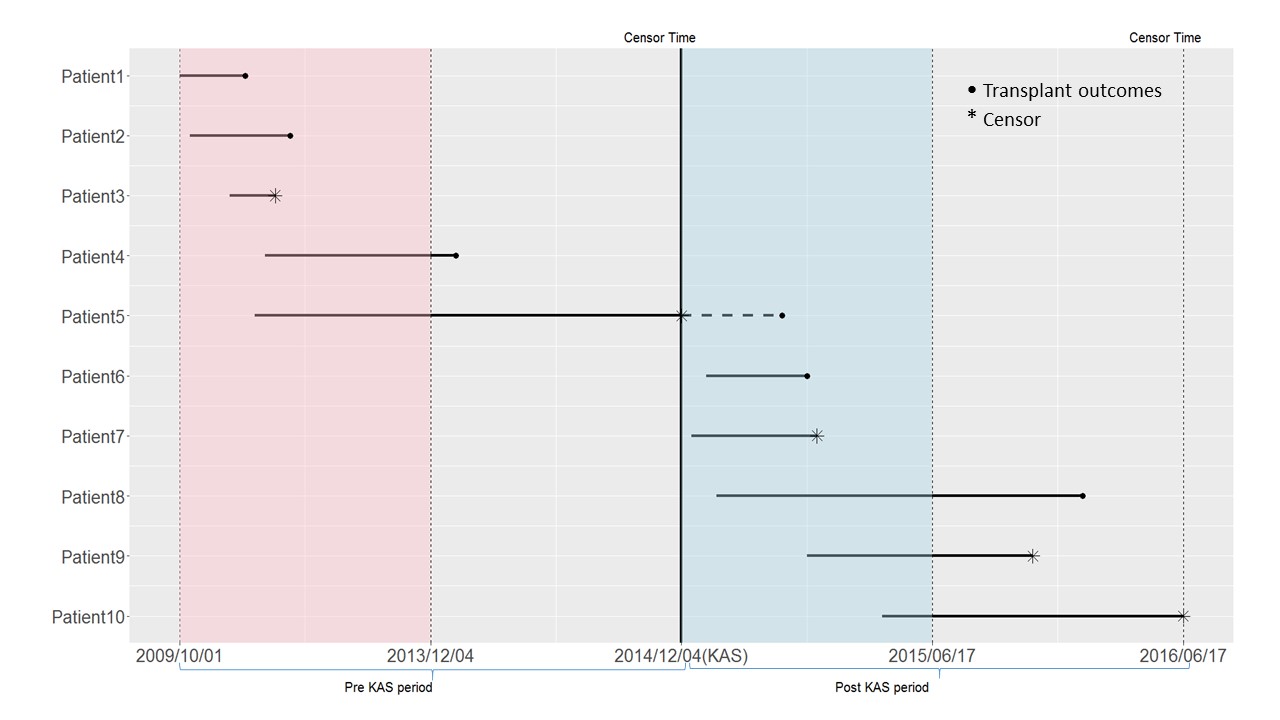


The pre-KAS cohort included patients who were registered between 10/1/2009 and 12/4/2013 (e.g., patients 1-5 above). The post-KAS cohort included patients who were registered between 12/4/2014 and 6/17/2015 (e.g., patients 6-10). Patients registered between 12/4/2013 and 12/3/2014 were not included, ensuring at least 1 of year follow up time for patients in both cohorts. Patients were followed until 6/17/2016, and patients who had no event before 6/17/2016 were censored on this date. Censoring could also be caused by loss to follow-up (e.g. patients 3, 7, and 9).

Some patients in the pre-KAS cohort had follow up time straddling both eras (e.g. patient 5, who registered in the pre-KAS era and had an event in the post-KAS era). To get the unbiased estimates of the probability of transplant outcomes prior to KAS implementation, we censored the follow-up time for them at KAS implementation. To estimate if KAS had an impact on patients who were listed pre-KAS but experienced no events in the pre-KAS era, we lifted censoring on these patients and re-estimated the probabilities. The resulting estimates of transplant probability reflect the influence of both the pre-KAS system and KAS. For example, assume we know that the 2-year transplant probabilities for highly sensitized patients are 0.2 pre-KAS and 0.4 after KAS implementation; that 100 patients completed 2 years’ follow up completely pre-KAS with 20 having transplants; and that another 100 had 2 years’ follow up straddling the two eras with 40 having transplants. After censoring the events occurring after KAS implementation, we would get an unbiased estimate of 0.2 for the pre-KAS cohort (the censoring mechanism assumes the 100 straddled patients would have had 20 events, so 40/200 = 0.2). However, if we don’t censor, we would get an estimate of (20+40)/(100+100) = 0.3;this is higher than 0.2 but lower than 0.4 because this estimate is for the pre-KAS cohort but also catches the impact of KAS.

**Panel B S1 Fig. Multi-State Model can be Viewed as a Series of Nested Competing Risk Models**

**
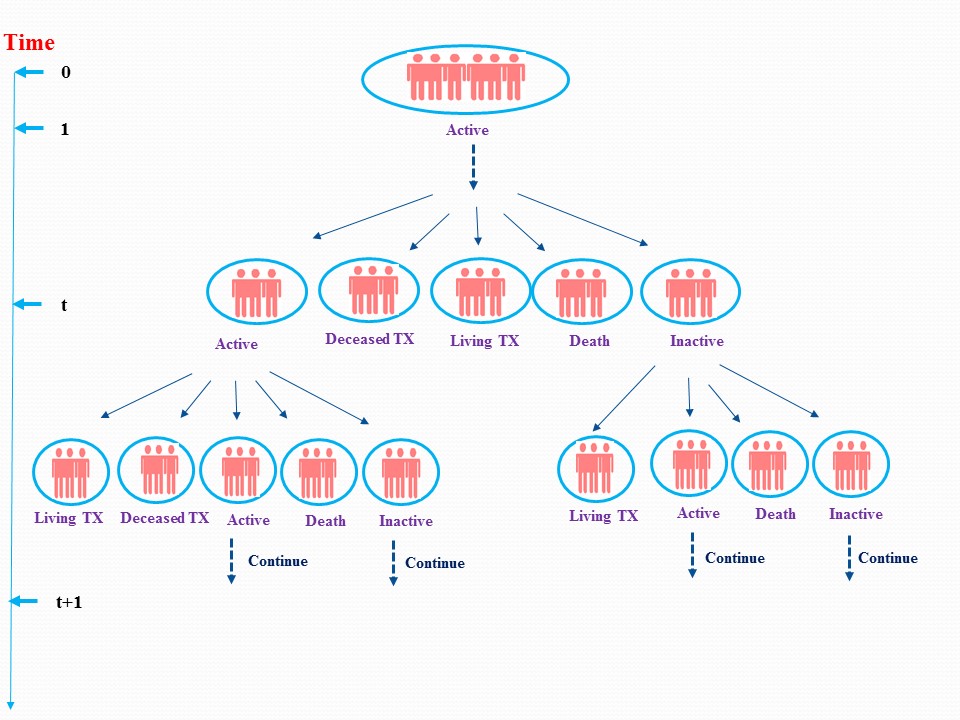
**

For example, consider a situation that all transplant candidates start with active status at the time of listing and some of them transition to intermediate inactive state or to one of the absorbing states at a random future time (t). Candidates from the initial state at time 0 have the risk of experiencing one of the competing events 1) inactive status; 2) living donor transplant; 3) deceased donor transplant; 4) death. For candidates who visit any of the absorbing states at time (t), there will be no further transition. Candidates, who are still in the initial sate at time (t), have the same set of competing risks. Candidates, who transition to inactive state at time (t), face another set of competing risks 1) active; 2) living donor transplant; 3) death. These series of competing risk experiments will be carried out until the observation period ends, or all candidates end up in the absorbing states or censored, whichever comes first.

**Panel C S1 Fig. Transition Hazard and Transition Probability**


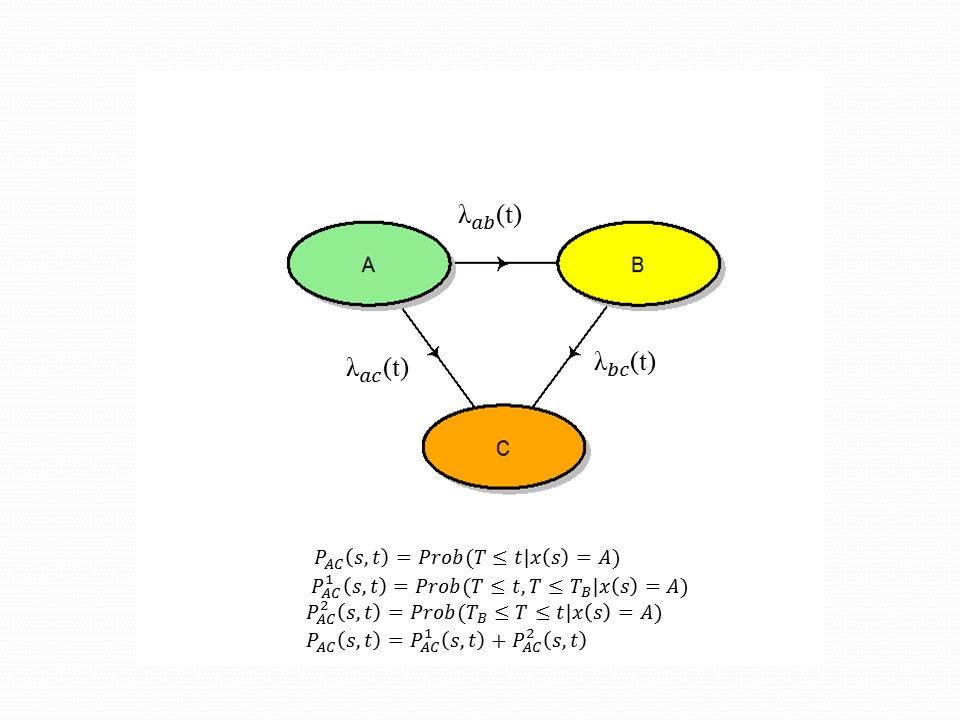


Transition hazard or intensity, analogous to the cause-specific hazard in the competing risk modeling, is the instantaneous transition risk from one state to another at a specific time. Cumulative transition hazard is the integrated transition hazard over a time interval. Transition probability, involving a longer time period, is the probability of transition from one state at time (s) to another at time (t), which can be estimated using the cumulative transition hazard matrix. Transition probability, usually involving two states and two time points, combines both direct and indirect transition probability from one state to another [15]. For example, using the example in figure s1b, the probability of transition from A to C is the sum of the probability of direct transition from A to C and the probability of indirect transition from A to C through B. Bearing this in mind, we would easily understand that why there is a proportion of transplant candidates with inactive status at the registration still get diseased donor transplant after some time into waiting process, even though patients with inactive status are not eligible for deceased donor transplant. It’s simply because that patients might have transitioned to active status before getting deceased donor transplant.
