# Supplementary material for: Transition probabilities between changing sensitization levels, waitlist activity status and competing-risk kidney transplant outcomes using multi-state modeling
Source: PLoS One. 2017 Dec 29;12(12):e0190277. doi: 10.1371/journal.pone.0190277 (PMC5747475; doi:10.1371/journal.pone.0190277)
Supplement: S2 Fig — (DOCX) [file pone.0190277.s002.docx]

**Supplemental information**

**S2 Fig. Dynamic Prediction of the Probability of Death at Year-3 in Pre-KAS Cohort**


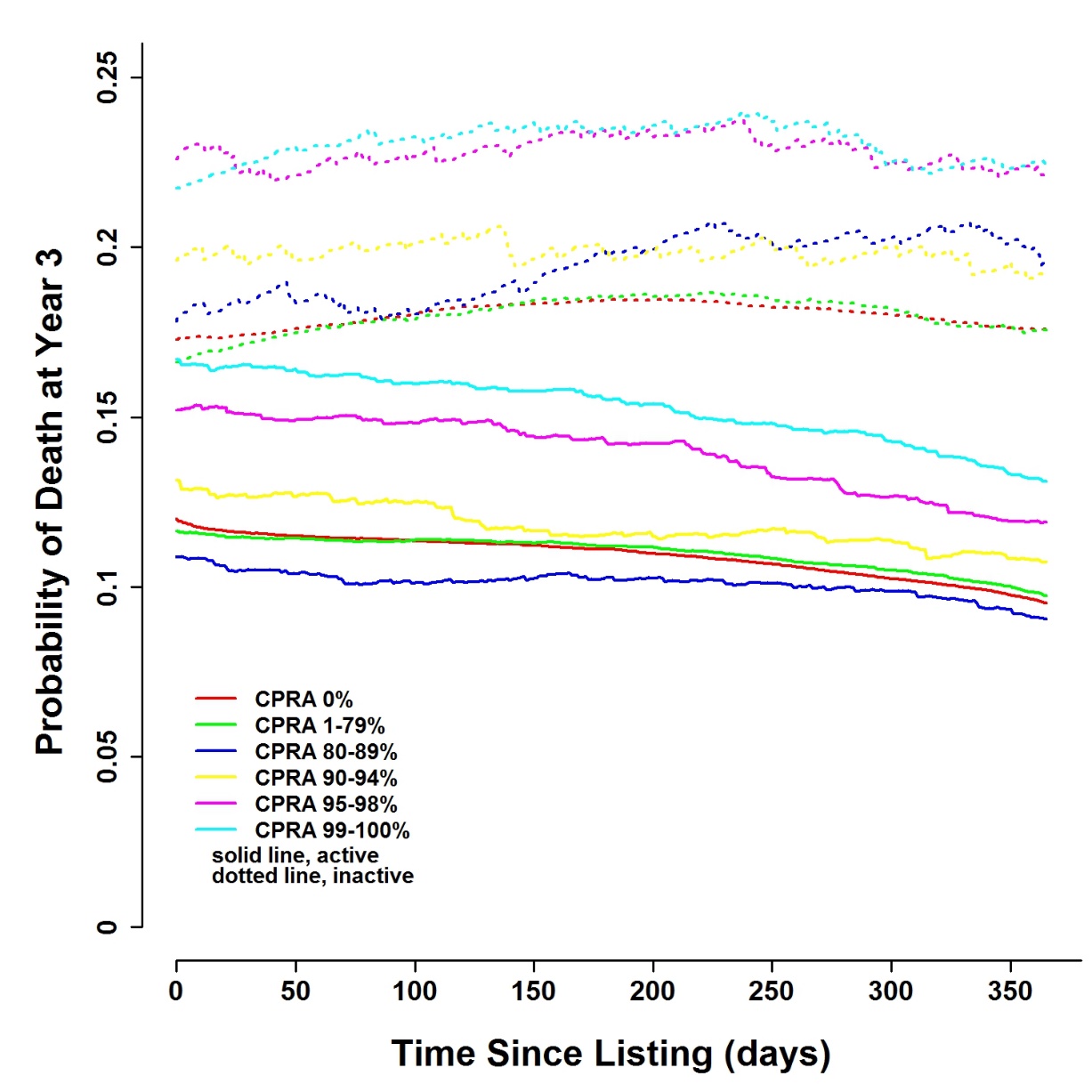


This figure shows the probability of death given the CPRA/activity status at any time of the first year of listing (or disease history within first year of listing). Patients with inactive status any time at first year of listing had increased probability of death at year 3. Probability estimate was included in table s2.
