# Supplementary material for: Transition probabilities between changing sensitization levels, waitlist activity status and competing-risk kidney transplant outcomes using multi-state modeling
Source: PLoS One. 2017 Dec 29;12(12):e0190277. doi: 10.1371/journal.pone.0190277 (PMC5747475; doi:10.1371/journal.pone.0190277)
Supplement: S3 Fig — (DOCX) [file pone.0190277.s003.docx]

**Supplemental information**

**S3 Fig. Dynamic Prediction of the Probability of Deceased Donor Transplant at Year-3 in Pre-KAS Cohort**


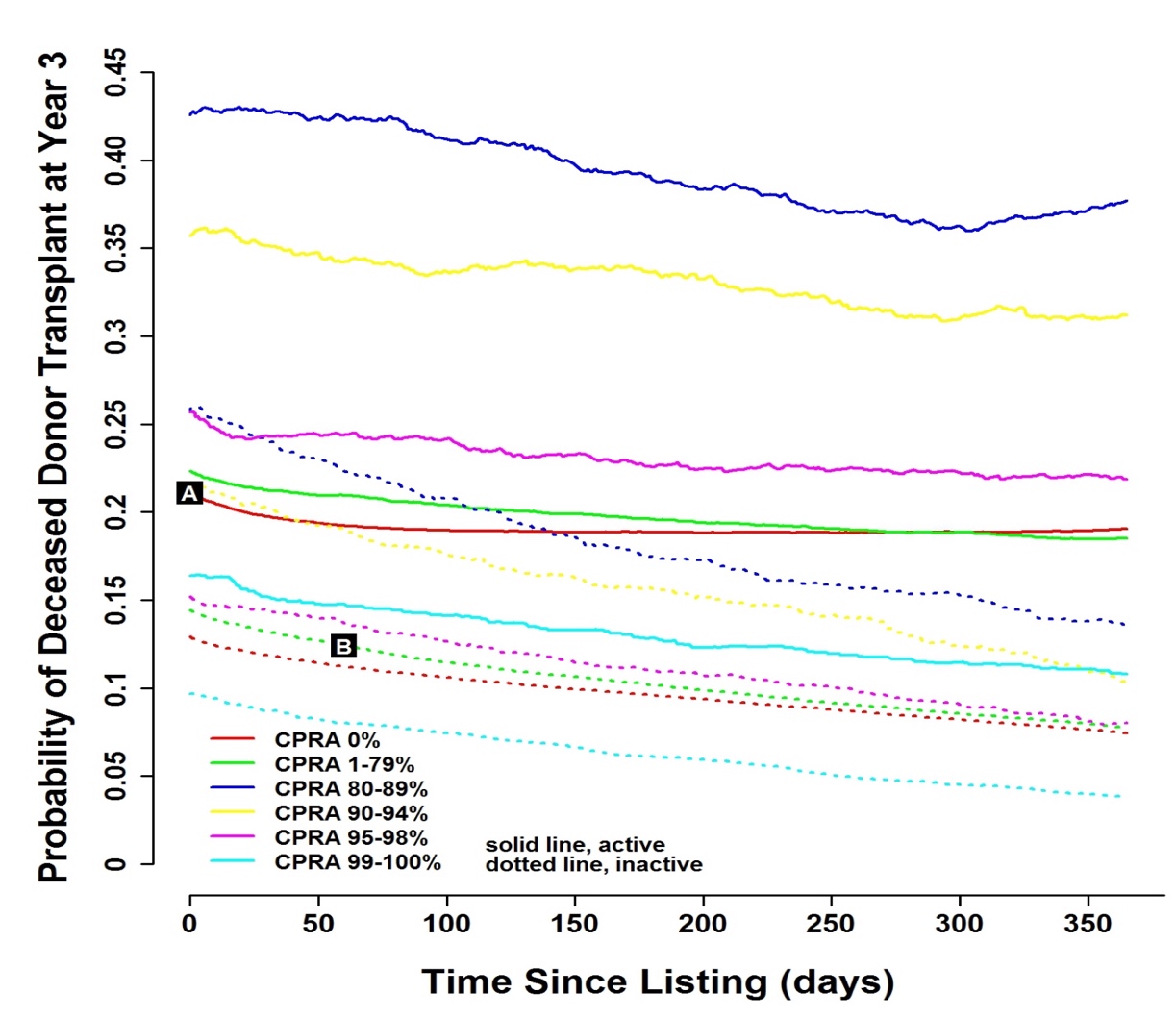


This figure shows the probability of deceased donor transplant at year 3 of listing given the CPRA/activity status at any time of the first year of listing (or disease history within first year of listing). For example, given the patient was listed as active with CPRA 0% at day 0, the predicted probability of transplant at year 3 was around 0.211 (point A), and if this patient transition to inactive with CPRA 1-79% at day 60, the predicted probability of transplant at year 3 deceased to 0.124 (point B). Probability estimate was included in S3 Table.
