# Supplementary material for: Transition probabilities between changing sensitization levels, waitlist activity status and competing-risk kidney transplant outcomes using multi-state modeling
Source: PLoS One. 2017 Dec 29;12(12):e0190277. doi: 10.1371/journal.pone.0190277 (PMC5747475; doi:10.1371/journal.pone.0190277)

**Supplemental information**

**Panel A S4 Fig. Line Graphs with 95% CI Showing Probabilities of Deceased Donor Transplant Between Whites, Hispanics and Blacks, Stratified by CPRA Category for Actively Listed Pre-KAS Individuals**

**
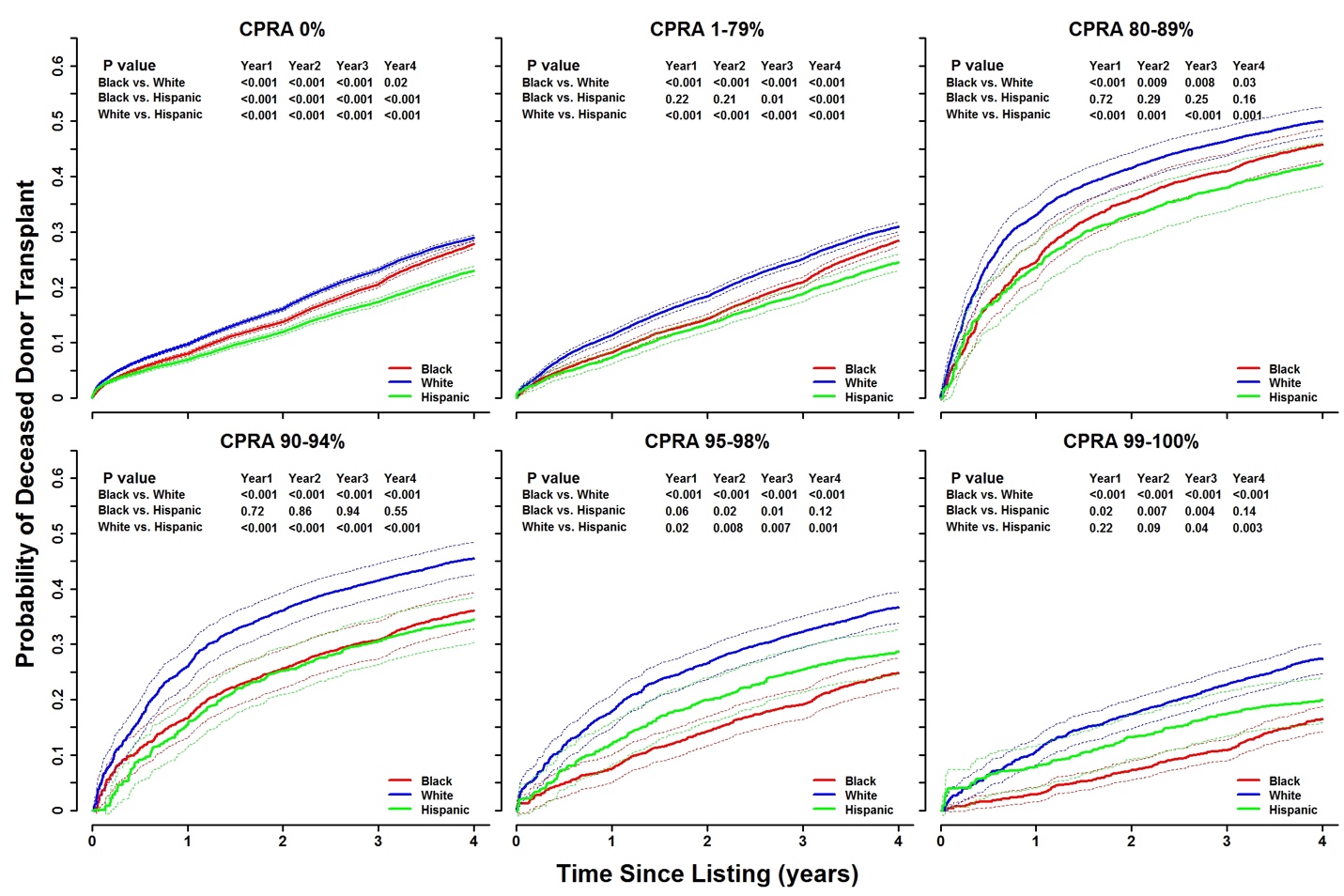
**

**Panel B S4 Fig. Line Graphs with 95% CI Showing Probabilities of Deceased Donor Transplant Between Whites, Hispanics and Blacks, Stratified by CPRA Category for Actively Listed Post-KAS Individuals**


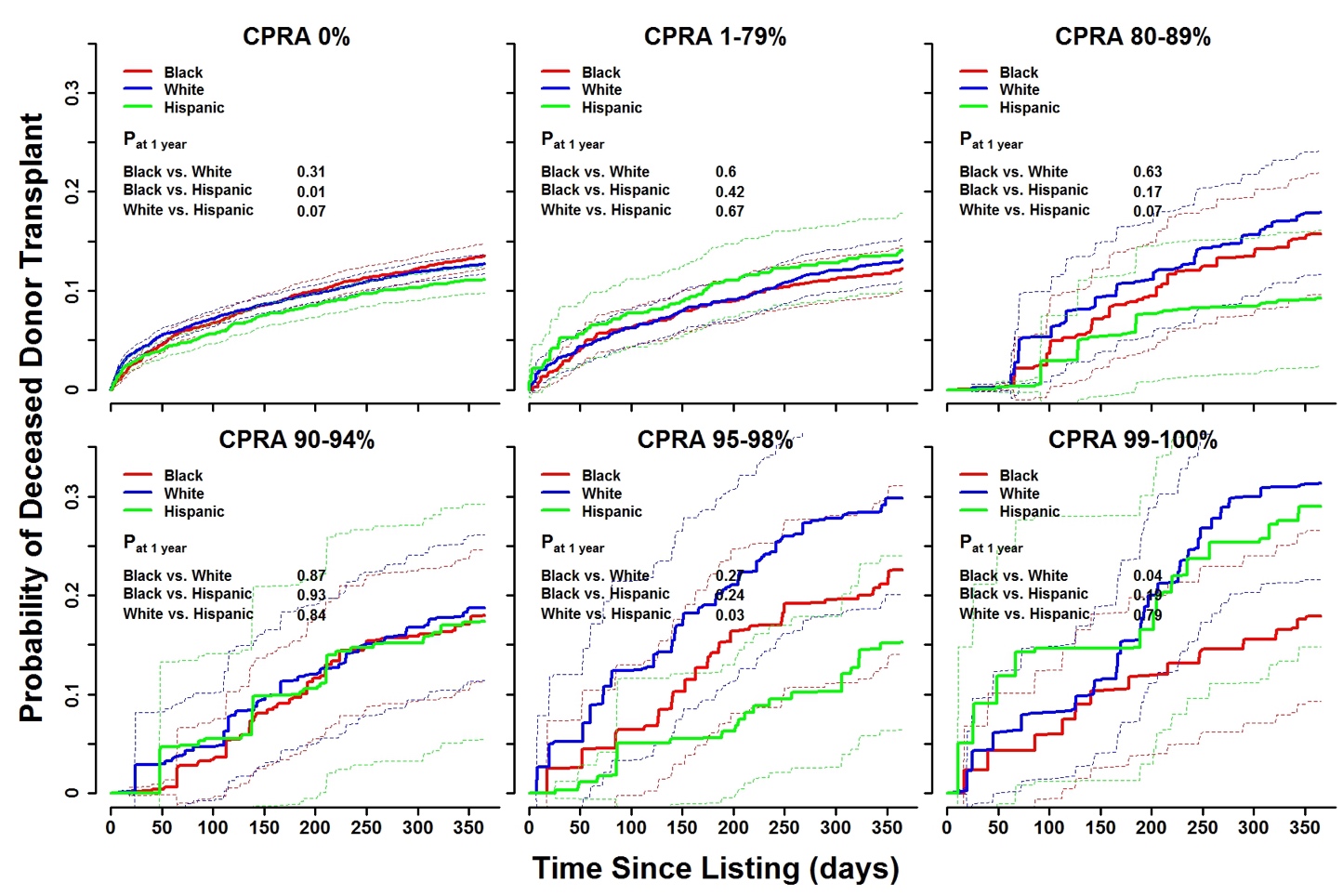

Supplement: S4 Fig — (Panel A) Line Graphs with 95% CI Showing Probabilities of Deceased Donor Transplant Between Whites, Hispanics and Blacks, Stratified by CPRA Category for Actively Listed Pre-KAS Individuals (Panel B) Line Graphs with 95% CI Showing Probabilities of Deceased Donor Transplant Between Whites, Hispanics and Blacks, Stratified by CPRA Category for Actively Listed Post-KAS Individuals. (DOCX) [file pone.0190277.s004.docx]
