# Supplementary material for: Transition probabilities between changing sensitization levels, waitlist activity status and competing-risk kidney transplant outcomes using multi-state modeling
Source: PLoS One. 2017 Dec 29;12(12):e0190277. doi: 10.1371/journal.pone.0190277 (PMC5747475; doi:10.1371/journal.pone.0190277)
Supplement: S1 Table — (DOCX) [file pone.0190277.s005.docx]

**Supplemental information**

**S1 Table. Probability of Death with 95% CI Predicted from Initial Status (Day 0 from listing)**

| **Cohort** | **Time**  **(days)** | **Active status** | **CPRA_0** | **CPRA1_79** | **CPRA80_89** | **CPRA90_94** | **CPRA95_98** | **CPRA99_100** |
| --- | --- | --- | --- | --- | --- | --- | --- | --- |
| Pre-KAS | 365 | active | 0.036 (0.035, 0.037) | 0.031 (0.029, 0.034) | 0.034 (0.027, 0.040) | 0.042 (0.032, 0.052) | 0.044 (0.035, 0.052) | 0.044 (0.034, 0.054) |
|  |  | inactive | 0.063 (0.060, 0.065) | 0.057 (0.053, 0.062) | 0.070 (0.054, 0.085) | 0.078 (0.057, 0.098) | 0.089 (0.068, 0.110) | 0.066 (0.050, 0.082) |
|  | 730 | active | 0.076 (0.075, 0.078) | 0.072 (0.069, 0.075) | 0.070 (0.063, 0.077) | 0.086 (0.075, 0.096) | 0.096 (0.086, 0.106) | 0.104 (0.091, 0.116) |
|  |  | inactive | 0.120 (0.117, 0.123) | 0.113 (0.107, 0.118) | 0.126 (0.109, 0.142) | 0.139 (0.118, 0.160) | 0.160 (0.138, 0.182) | 0.145 (0.126, 0.165) |
|  | 1095 | active | 0.120 (0.118, 0.122) | 0.117 (0.113, 0.120) | 0.109 (0.101, 0.117) | 0.132 (0.120, 0.143) | 0.152 (0.140, 0.164) | 0.167 (0.152, 0.182) |
|  |  | inactive | 0.173 (0.170, 0.176) | 0.166 (0.160, 0.172) | 0.178 (0.162, 0.195) | 0.196 (0.175, 0.217) | 0.226 (0.204, 0.248) | 0.217 (0.197, 0.238) |
|  | 1460 | active | 0.160 (0.157, 0.163) | 0.157 (0.153, 0.162) | 0.142 (0.133, 0.150) | 0.171 (0.159, 0.183) | 0.199 (0.186, 0.212) | 0.223 (0.206, 0.240) |
|  |  | inactive | 0.218 (0.215, 0.222) | 0.212 (0.205, 0.218) | 0.220 (0.203, 0.236) | 0.243 (0.222, 0.264) | 0.276 (0.255, 0.298) | 0.277 (0.255, 0.299) |
| Post-KAS | 365 | active | 0.033 (0.030, 0.036) | 0.028 (0.022, 0.034) | 0.029 (0.015, 0.043) | 0.034 (0.016, 0.052) | 0.027 (0.013, 0.042) | 0.051 (0.020, 0.081) |
|  |  | inactive | 0.059 (0.052, 0.065) | 0.058 (0.044, 0.071) | 0.091 (0.027, 0.154) | 0.079 (0.009, 0.149) | 0.099 (0.016, 0.181) | 0.056 (0.013, 0.099) |
