# Supplementary material for: Transition probabilities between changing sensitization levels, waitlist activity status and competing-risk kidney transplant outcomes using multi-state modeling
Source: PLoS One. 2017 Dec 29;12(12):e0190277. doi: 10.1371/journal.pone.0190277 (PMC5747475; doi:10.1371/journal.pone.0190277)
Supplement: S2 Table — (DOCX) [file pone.0190277.s006.docx]

**Supplemental information**

**S2 Table. Dynamic Prediction of the Probability of Death at Year-3 in Pre-KAS Cohort, Given Disease History within Year-1 of Listing**

| **Time**  **(days)** | **Active CPRA_0** | **Active CPRA 1_79** | **Active CPRA 80_89** | **Active CPRA90_94** | **Active CPRA95_98** | **Active CPRA99_100** |
| --- | --- | --- | --- | --- | --- | --- |
| 0 | 0.120 (0.118, 0.122) | 0.117 (0.113, 0.120) | 0.109 (0.101, 0.117) | 0.132 (0.120, 0.143) | 0.152 (0.140, 0.164) | 0.167 (0.152, 0.182) |
| 30 | 0.116 (0.114, 0.118) | 0.115 (0.111, 0.118) | 0.105 (0.097, 0.113) | 0.126 (0.116, 0.137) | 0.151 (0.139, 0.163) | 0.165 (0.151, 0.180) |
| 60 | 0.115 (0.112, 0.117) | 0.114 (0.110, 0.118) | 0.104 (0.096, 0.112) | 0.128 (0.117, 0.138) | 0.150 (0.138, 0.162) | 0.162 (0.147, 0.177) |
| 90 | 0.114 (0.111, 0.116) | 0.113 (0.110, 0.117) | 0.102 (0.094, 0.110) | 0.125 (0.115, 0.136) | 0.148 (0.136, 0.160) | 0.161 (0.146, 0.175) |
| 120 | 0.113 (0.111, 0.116) | 0.114 (0.110, 0.118) | 0.102 (0.094, 0.110) | 0.120 (0.109, 0.130) | 0.148 (0.136, 0.161) | 0.160 (0.145, 0.175) |
| 150 | 0.112 (0.110, 0.115) | 0.113 (0.109, 0.117) | 0.103 (0.094, 0.111) | 0.116 (0.106, 0.127) | 0.144 (0.132, 0.157) | 0.158 (0.143, 0.173) |
| 180 | 0.111 (0.108, 0.114) | 0.112 (0.108, 0.116) | 0.103 (0.094, 0.111) | 0.115 (0.105, 0.126) | 0.143 (0.131, 0.156) | 0.155 (0.140, 0.170) |
| 210 | 0.109 (0.107, 0.112) | 0.111 (0.107, 0.115) | 0.102 (0.093, 0.110) | 0.116 (0.105, 0.126) | 0.143 (0.130, 0.156) | 0.151 (0.137, 0.166) |
| 240 | 0.107 (0.105, 0.110) | 0.109 (0.105, 0.113) | 0.101 (0.093, 0.110) | 0.116 (0.105, 0.127) | 0.135 (0.123, 0.148) | 0.148 (0.133, 0.163) |
| 270 | 0.105 (0.102, 0.108) | 0.107 (0.103, 0.111) | 0.100 (0.091, 0.109) | 0.115 (0.103, 0.126) | 0.132 (0.119, 0.145) | 0.146 (0.131, 0.161) |
| 300 | 0.102 (0.100, 0.105) | 0.105 (0.101, 0.109) | 0.099 (0.090, 0.108) | 0.114 (0.102, 0.126) | 0.127 (0.114, 0.139) | 0.143 (0.128, 0.158) |
| 330 | 0.100 (0.097, 0.103) | 0.102 (0.098, 0.106) | 0.096 (0.087, 0.106) | 0.110 (0.098, 0.123) | 0.122 (0.109, 0.134) | 0.138 (0.123, 0.153) |
| 360 | 0.096 (0.094, 0.099) | 0.098 (0.094, 0.103) | 0.091 (0.082, 0.101) | 0.108 (0.095, 0.121) | 0.119 (0.107, 0.132) | 0.132 (0.117, 0.147) |
| **Time**  **(days)** | **Inactive CPRA0** | **Inactive CPRA1_79** | **Inactive CPRA80_89** | **Inactive CPRA90_94** | **Inactive CPRA95_98** | **Inactive CPRA99_100** |
| 0 | 0.173 (0.170, 0.176) | 0.166 (0.160, 0.172) | 0.178 (0.162, 0.195) | 0.196 (0.175, 0.217) | 0.226 (0.204, 0.248) | 0.217 (0.197, 0.238) |
| 30 | 0.174 (0.171, 0.178) | 0.172 (0.166, 0.178) | 0.184 (0.166, 0.201) | 0.195 (0.175, 0.216) | 0.223 (0.201, 0.246) | 0.225 (0.203, 0.247) |
| 60 | 0.177 (0.173, 0.181) | 0.176 (0.169, 0.183) | 0.186 (0.168, 0.205) | 0.197 (0.176, 0.218) | 0.224 (0.202, 0.247) | 0.230 (0.207, 0.253) |
| 90 | 0.179 (0.175, 0.183) | 0.179 (0.172, 0.186) | 0.180 (0.162, 0.198) | 0.201 (0.178, 0.224) | 0.226 (0.203, 0.248) | 0.231 (0.207, 0.255) |
| 120 | 0.182 (0.178, 0.187) | 0.181 (0.174, 0.188) | 0.183 (0.165, 0.201) | 0.204 (0.180, 0.228) | 0.227 (0.204, 0.250) | 0.233 (0.209, 0.258) |
| 150 | 0.183 (0.179, 0.188) | 0.184 (0.177, 0.192) | 0.190 (0.170, 0.209) | 0.197 (0.174, 0.220) | 0.232 (0.207, 0.256) | 0.237 (0.212, 0.262) |
| 180 | 0.185 (0.180, 0.189) | 0.185 (0.178, 0.193) | 0.199 (0.178, 0.220) | 0.199 (0.175, 0.223) | 0.234 (0.210, 0.259) | 0.235 (0.210, 0.260) |
| 210 | 0.185 (0.180, 0.189) | 0.186 (0.178, 0.194) | 0.203 (0.181, 0.226) | 0.199 (0.174, 0.223) | 0.233 (0.208, 0.258) | 0.234 (0.209, 0.259) |
| 240 | 0.183 (0.178, 0.187) | 0.186 (0.178, 0.194) | 0.203 (0.180, 0.225) | 0.200 (0.175, 0.226) | 0.235 (0.209, 0.262) | 0.239 (0.212, 0.265) |
| 270 | 0.182 (0.177, 0.186) | 0.184 (0.176, 0.192) | 0.202 (0.179, 0.224) | 0.195 (0.170, 0.220) | 0.231 (0.205, 0.258) | 0.236 (0.209, 0.263) |
| 300 | 0.180 (0.175, 0.185) | 0.182 (0.174, 0.190) | 0.203 (0.180, 0.226) | 0.200 (0.173, 0.227) | 0.225 (0.199, 0.252) | 0.225 (0.199, 0.251) |
| 330 | 0.178 (0.173, 0.183) | 0.177 (0.169, 0.185) | 0.207 (0.182, 0.231) | 0.197 (0.170, 0.225) | 0.223 (0.196, 0.250) | 0.225 (0.198, 0.251) |
| 360 | 0.176 (0.171, 0.181) | 0.176 (0.167, 0.184) | 0.200 (0.175, 0.224) | 0.191 (0.163, 0.218) | 0.223 (0.195, 0.250) | 0.225 (0.198, 0.252) |
