# Supplementary material for: Transition probabilities between changing sensitization levels, waitlist activity status and competing-risk kidney transplant outcomes using multi-state modeling
Source: PLoS One. 2017 Dec 29;12(12):e0190277. doi: 10.1371/journal.pone.0190277 (PMC5747475; doi:10.1371/journal.pone.0190277)
Supplement: S3 Table — (DOCX) [file pone.0190277.s007.docx]

**Supplemental information**

**S3 Table. Probability of Deceased Donor Transplant with 95% CI Predicted from Day 0 of Listing**

| **Cohort** | **Time**  **(days)** | **Activity status** | **Censoring^§^** | **CPRA_0** | **CPRA1_79** | **CPRA80_89** | **CPRA90_94** | **CPRA95_98** | **CPRA99_100** |
| --- | --- | --- | --- | --- | --- | --- | --- | --- | --- |
| Pre-KAS | 365 | active | censored | 0.084 (0.082, 0.086) | 0.093  (0.089, 0.098) | 0.280  (0.261, 0.300) | 0.208  (0.187, 0.229) | 0.127  (0.110, 0.144) | 0.067  (0.054, 0.080) |
|  |  |  | Not censored | 0.084  (0.082, 0.086) | 0.093  (0.089, 0.098) | 0.280  (0.261, 0.300) | 0.208  (0.187, 0.229) | 0.127 (0.110, 0.144) | 0.067 (0.054, 0.080) |
|  |  | inactive | censored | 0.026 (0.025, 0.026) | 0.034 (0.032, 0.036) | 0.113 (0.100, 0.125) | 0.078 (0.067, 0.089) | 0.044 (0.036, 0.051) | 0.023 (0.018, 0.027) |
|  |  |  | Not censored | 0.026 (0.025, 0.026) | 0.034 (0.032, 0.036) | 0.113 (0.100, 0.125) | 0.078 (0.067, 0.089) | 0.044 (0.036, 0.051) | 0.023 (0.018, 0.027) |
|  | 730 | active | censored | 0.144 (0.141, 0.146) | 0.158 (0.153, 0.163) | 0.375 (0.356, 0.393) | 0.304 (0.283, 0.324) | 0.204 (0.187, 0.222) | 0.120 (0.106, 0.134) |
|  |  |  | Not censored | 0.145 (0.143, 0.147) | 0.159 (0.154, 0.164) | 0.374 (0.356, 0.392) | 0.306 (0.286, 0.326) | 0.212 (0.194, 0.229) | 0.134 (0.120, 0.149) |
|  |  | inactive | censored | 0.069 (0.068, 0.071) | 0.084 (0.081, 0.087) | 0.201 (0.186, 0.216) | 0.159 (0.144, 0.174) | 0.103 (0.093, 0.114) | 0.060 (0.053, 0.068) |
|  |  |  | Not censored | 0.070 (0.069, 0.072) | 0.085 (0.082, 0.087) | 0.199 (0.185, 0.214) | 0.161 (0.146, 0.176) | 0.109 (0.099, 0.120) | 0.072 (0.064, 0.080) |
|  | 1095 | active | censored | 0.211 (0.208, 0.214) | 0.223 (0.218, 0.229) | 0.426 (0.409, 0.443) | 0.357 (0.338, 0.377) | 0.257 (0.240, 0.274) | 0.164 (0.149, 0.179) |
|  |  |  | Not censored | 0.206 (0.203, 0.209) | 0.220 (0.215, 0.225) | 0.425 (0.408, 0.442) | 0.364 (0.345, 0.383) | 0.275 (0.258, 0.292) | 0.200 (0.186, 0.215) |
|  |  | inactive | censored | 0.129 (0.127, 0.132) | 0.144 (0.140, 0.148) | 0.258 (0.243, 0.273) | 0.213 (0.196, 0.229) | 0.152 (0.140, 0.164) | 0.097 (0.087, 0.106) |
|  |  |  | Not censored | 0.124 (0.122, 0.127) | 0.141 (0.137, 0.144) | 0.254 (0.239, 0.269) | 0.218 (0.202, 0.234) | 0.167 (0.155, 0.179) | 0.130 (0.119, 0.140) |
|  | 1460 | active | censored | 0.273 (0.270, 0.277) | 0.287 (0.281, 0.293) | 0.468 (0.451, 0.484) | 0.403 (0.384, 0.421) | 0.304 (0.287, 0.322) | 0.210 (0.195, 0.226) |
|  |  |  | Not censored | 0.259 (0.256, 0.262) | 0.274 (0.269, 0.279) | 0.464 (0.447, 0.480) | 0.408 (0.390, 0.426) | 0.326 (0.309, 0.342) | 0.256 (0.241, 0.271) |
|  |  | inactive | censored | 0.190 (0.187, 0.194) | 0.208 (0.203, 0.213) | 0.305 (0.290, 0.321) | 0.260 (0.243, 0.277) | 0.198 (0.184, 0.212) | 0.139 (0.127, 0.150) |
|  |  |  | Not censored | 0.176 (0.174, 0.179) | 0.194 (0.190, 0.199) | 0.298 (0.282, 0.313) | 0.265 (0.248, 0.281) | 0.216 (0.203, 0.230) | 0.182 (0.170, 0.194) |
| Post-KAS | 365 | active | NA | 0.123 (0.117, 0.129) | 0.125 (0.112, 0.139) | 0.152 (0.116, 0.189) | 0.180 (0.137, 0.223) | 0.242 (0.188, 0.295) | 0.252 (0.195, 0.308) |
|  | 365 | inactive |  | 0.037 (0.035, 0.040) | 0.040 (0.035, 0.046) | 0.065 (0.042, 0.088) | 0.089 (0.058, 0.120) | 0.099 (0.064, 0.134) | 0.104 (0.065, 0.143) |

**^§^**Two sets of analyses were performed to evaluate the impact of KAS. First, transplant probabilities were estimated separately in pre- and post-KAS cohorts and outcomes for candidates listed pre-KAS were censored at KAS implementation. Second, to assess the impact of KAS on candidates listed pre-KAS, who did not experience an outcome in the pre-KAS era, we lifted right-censoring at KAS implementation and continued to measure their outcomes in the post-KAS period.
