# Supplementary material for: Transition probabilities between changing sensitization levels, waitlist activity status and competing-risk kidney transplant outcomes using multi-state modeling
Source: PLoS One. 2017 Dec 29;12(12):e0190277. doi: 10.1371/journal.pone.0190277 (PMC5747475; doi:10.1371/journal.pone.0190277)
Supplement: S4 Table — (DOCX) [file pone.0190277.s008.docx]

**Supplemental information**

**S4 Table: Dynamic Prediction of the Probability of Deceased Donor Transplant at Year-3 in Pre-KAS Cohort, Given Disease History within First Year of Listing**

| **Deceased donor transplant** | | | | | | |
| --- | --- | --- | --- | --- | --- | --- |
| **Time**  **(days)** | **Active CPRA0** | **Active CPRA1_79** | **Active CPRA80_89** | **Active CPRA90_94** | **Active CPRA95_98** | **Active CPRA99_100** |
| 0 | 0.211 (0.208, 0.214) | 0.223 (0.218, 0.229) | 0.426 (0.409, 0.443) | 0.357 (0.338, 0.377) | 0.257 (0.240, 0.274) | 0.164 (0.149, 0.179) |
| 30 | 0.198 (0.194, 0.201) | 0.213 (0.207, 0.218) | 0.427 (0.409, 0.444) | 0.351 (0.332, 0.370) | 0.243 (0.227, 0.260) | 0.152 (0.138, 0.165) |
| 60 | 0.192 (0.189, 0.196) | 0.209 (0.204, 0.215) | 0.424 (0.407, 0.442) | 0.342 (0.323, 0.361) | 0.244 (0.228, 0.261) | 0.147 (0.134, 0.161) |
| 90 | 0.190 (0.187, 0.193) | 0.205 (0.200, 0.211) | 0.417 (0.399, 0.435) | 0.335 (0.316, 0.354) | 0.242 (0.226, 0.259) | 0.143 (0.129, 0.156) |
| 120 | 0.189 (0.186, 0.193) | 0.201 (0.196, 0.207) | 0.409 (0.391, 0.428) | 0.339 (0.319, 0.358) | 0.236 (0.219, 0.252) | 0.138 (0.124, 0.151) |
| 150 | 0.188 (0.185, 0.192) | 0.199 (0.194, 0.204) | 0.398 (0.380, 0.416) | 0.338 (0.318, 0.358) | 0.233 (0.216, 0.249) | 0.133 (0.120, 0.146) |
| 180 | 0.189 (0.185, 0.192) | 0.196 (0.190, 0.201) | 0.389 (0.371, 0.407) | 0.336 (0.316, 0.356) | 0.226 (0.209, 0.242) | 0.128 (0.116, 0.141) |
| 210 | 0.188 (0.185, 0.192) | 0.194 (0.188, 0.199) | 0.385 (0.367, 0.404) | 0.328 (0.307, 0.348) | 0.223 (0.207, 0.240) | 0.124 (0.111, 0.136) |
| 240 | 0.189 (0.185, 0.192) | 0.192 (0.186, 0.197) | 0.374 (0.355, 0.392) | 0.324 (0.303, 0.345) | 0.226 (0.208, 0.243) | 0.122 (0.109, 0.135) |
| 270 | 0.188 (0.185, 0.192) | 0.189 (0.184, 0.195) | 0.370 (0.351, 0.389) | 0.314 (0.293, 0.335) | 0.223 (0.206, 0.241) | 0.117 (0.105, 0.130) |
| 300 | 0.189 (0.185, 0.193) | 0.188 (0.183, 0.194) | 0.363 (0.343, 0.382) | 0.310 (0.289, 0.332) | 0.222 (0.205, 0.240) | 0.115 (0.102, 0.127) |
| 330 | 0.189 (0.185, 0.193) | 0.186 (0.180, 0.191) | 0.368 (0.347, 0.388) | 0.311 (0.288, 0.333) | 0.221 (0.203, 0.239) | 0.112 (0.099, 0.125) |
| 360 | 0.190 (0.186, 0.194) | 0.185 (0.179, 0.191) | 0.374 (0.353, 0.396) | 0.311 (0.287, 0.334) | 0.219 (0.200, 0.238) | 0.108 (0.095, 0.121) |
| **Time**  **(Days)** | **Inactive CPRA0** | **Inactive CPRA1_79** | **Inactive CPRA80_89** | **Inactive CPRA90_94** | **Inactive CPRA95_98** | **Inactive CPRA99_100** |
| 0 | 0.129 (0.127, 0.132) | 0.144 (0.140, 0.148) | 0.258 (0.243, 0.273) | 0.213 (0.196, 0.229) | 0.152 (0.140, 0.164) | 0.097 (0.087, 0.106) |
| 30 | 0.119 (0.116, 0.121) | 0.132 (0.128, 0.136) | 0.241 (0.226, 0.256) | 0.203 (0.187, 0.218) | 0.144 (0.132, 0.156) | 0.087 (0.078, 0.096) |
| 60 | 0.112 (0.110, 0.115) | 0.124 (0.121, 0.128) | 0.223 (0.208, 0.238) | 0.190 (0.175, 0.206) | 0.137 (0.125, 0.149) | 0.080 (0.071, 0.089) |
| 90 | 0.108 (0.105, 0.110) | 0.117 (0.113, 0.120) | 0.210 (0.195, 0.225) | 0.179 (0.164, 0.195) | 0.129 (0.118, 0.141) | 0.076 (0.067, 0.084) |
| 120 | 0.103 (0.101, 0.105) | 0.111 (0.107, 0.115) | 0.200 (0.185, 0.215) | 0.168 (0.152, 0.183) | 0.122 (0.110, 0.133) | 0.071 (0.063, 0.079) |
| 150 | 0.099 (0.097, 0.102) | 0.107 (0.103, 0.110) | 0.186 (0.171, 0.200) | 0.163 (0.147, 0.178) | 0.115 (0.103, 0.126) | 0.066 (0.058, 0.074) |
| 180 | 0.096 (0.094, 0.098) | 0.102 (0.098, 0.106) | 0.175 (0.160, 0.189) | 0.156 (0.141, 0.172) | 0.110 (0.099, 0.121) | 0.061 (0.054, 0.069) |
| 210 | 0.092 (0.090, 0.095) | 0.098 (0.094, 0.101) | 0.168 (0.154, 0.183) | 0.149 (0.134, 0.164) | 0.107 (0.096, 0.119) | 0.058 (0.051, 0.065) |
| 240 | 0.089 (0.087, 0.091) | 0.093 (0.089, 0.096) | 0.159 (0.145, 0.173) | 0.142 (0.127, 0.158) | 0.101 (0.090, 0.112) | 0.052 (0.045, 0.059) |
| 270 | 0.085 (0.083, 0.088) | 0.089 (0.086, 0.093) | 0.155 (0.141, 0.170) | 0.138 (0.122, 0.153) | 0.097 (0.086, 0.107) | 0.048 (0.041, 0.055) |
| 300 | 0.082 (0.080, 0.084) | 0.085 (0.082, 0.089) | 0.153 (0.138, 0.167) | 0.124 (0.109, 0.139) | 0.091 (0.080, 0.101) | 0.045 (0.039, 0.052) |
| 330 | 0.079 (0.076, 0.081) | 0.082 (0.078, 0.085) | 0.140 (0.126, 0.155) | 0.117 (0.102, 0.132) | 0.086 (0.075, 0.096) | 0.042 (0.036, 0.048) |
| 360 | 0.075 (0.073, 0.077) | 0.078 (0.075, 0.081) | 0.137 (0.123, 0.151) | 0.107 (0.092, 0.121) | 0.080 (0.070, 0.090) | 0.038 (0.033, 0.044) |
